# Beyond the average: An updated framework for understanding the relationship between cell growth, DNA replication, and division in a bacterial system

**DOI:** 10.1371/journal.pgen.1010505

**Published:** 2023-01-05

**Authors:** Sara Sanders, Kunaal Joshi, Petra Anne Levin, Srividya Iyer-Biswas

**Affiliations:** 1 Department of Biology, Washington University in St. Louis, St. Louis, Missouri, United States of America; 2 Department of Physics and Astronomy, Purdue University, West Lafayette, Indiana, United States of America; 3 Santa Fe Institute, Santa Fe, New Mexico, United States of America; The University of North Carolina at Chapel Hill, UNITED STATES

## Abstract

Our understanding of the bacterial cell cycle is framed largely by population-based experiments that focus on the behavior of idealized average cells. Most famously, the contributions of Cooper and Helmstetter help to contextualize the phenomenon of overlapping replication cycles observed in rapidly growing bacteria. Despite the undeniable value of these approaches, their necessary reliance on the behavior of idealized average cells masks the stochasticity inherent in single-cell growth and physiology and limits their mechanistic value. To bridge this gap, we propose an updated and agnostic framework, informed by extant single-cell data, that quantitatively accounts for stochastic variations in single-cell dynamics and the impact of medium composition on cell growth and cell cycle progression. In this framework, stochastic timers sensitive to medium composition impact the relationship between cell cycle events, accounting for observed differences in the relationship between cell cycle events in slow- and fast-growing cells. We conclude with a roadmap for potential application of this framework to longstanding open questions in the bacterial cell cycle field.

## Introduction

Proliferation of organisms across the tree of life requires effective coordination of cell growth, DNA replication, and division. Coordination is challenging in bacteria for which population mass doubling times can vary as much as 5-fold with nutrient availability. In many bacteria, including the model organism *Escherichia coli*, the time required to complete a round of DNA replication can be longer than the mass doubling time, particularly under nutrient rich conditions, resulting in multiple ongoing cycles of DNA replication on the same chromosomal template [1,2].

Traditionally, bacteriologists have relied on population-level strategies to understand fundamental aspects of bacterial physiology. Population-level approaches that describe the behavior of idealized average cells, serve as the foundation for prevailing models of bacterial growth, size, and cell cycle regulation.

Advances in microfluidics and single-cell analysis, however, reveal disconnects between population-based behaviors and the reality in single cells. Most importantly, it is now clear that population-level analysis masks stochastic, cell-to-cell variations in growth rate, size at division, and the timing of cell cycle events, resulting in models that do not always hold up in single cells [3–9].

Additionally, despite the precision and care earlier investigators took to emphasize the phenomenological nature of their models, the relationships they describe are often misinterpreted as determinant. Growth rate, in particular, is often portrayed as a determinant variable with regard to the relationship between cell cycle events despite its inherently complex nature, a feature noted by pioneers including Ole Maaløe, Moselio Schaechter, Charles Helmstetter, Stephen Cooper, and Frederick Neidhart [1,10].

Here, we review the prevailing population-based models of bacterial growth and cell cycle progression, highlighting the core reasoning underlying each. Next, we leverage extant data to propose a framework from which to understand the bacterial cell cycle accounting for physiology and stochasticity inherent in single cells. Finally, we end with a discussion of open questions and avenues for future research.

## The nutrient growth law

Early work on the *E*. *coli* cell cycle focused on the relationships between growth rate and 4 parameters: cell size, RNA content, DNA content, and nutrient composition. In their classic 1958 study, Schaechter, Maaløe, and Kjeldgaard observed that the average mass of *Salmonella* Typhimurium increases exponentially with nutrient-imposed increases in population mass doubling time [11]. Protein, RNA, and DNA content similarly increase, indicating that their overall concentration (mass/mass) remains constant.

**Fig 1:**
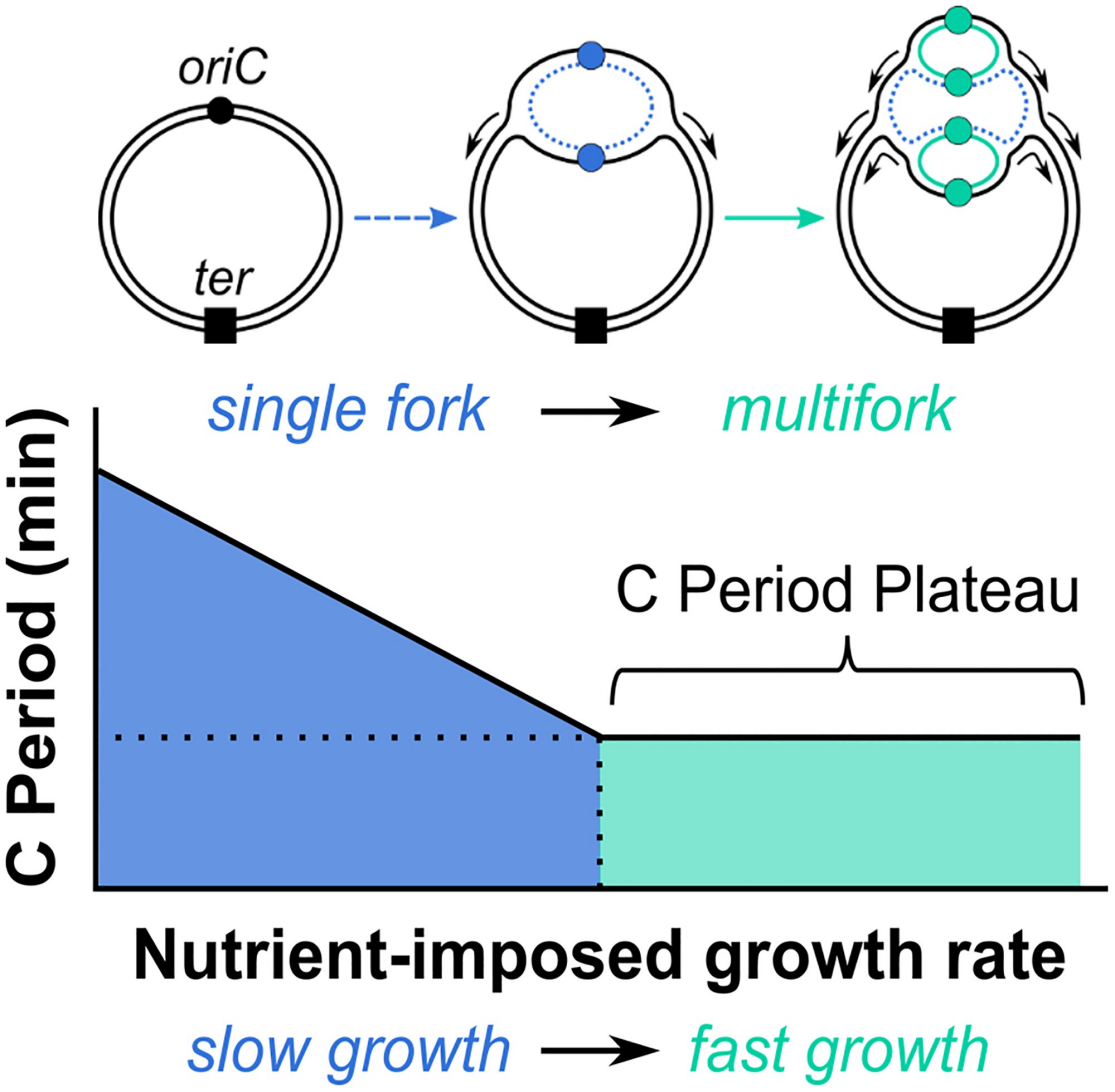
Replication of the *E*. *coli* chromosome. Top: Bacterial chromosome depicting the origin of replication (*oriC*, ●) and terminus *(ter*, ■). After birth, replication initiation yields a single replication bubble, a replication state termed “single fork replication” (- - -). When a second initiation event occurs before termination of the prior round, “multifork replication” occurs (—). Active origins and newly synthesized DNA are indicated with colors corresponding to replication state. Bottom: In the Cooper–Helmstetter model, as nutrient-imposed growth rate increases, the C period length decreases until it reaches a plateau during fast growth.

Although the data are noisy (see Figure 1 of reference 11, for example, related to size), the best-fit line drawn through over 20 different media conditions suggests a simplified model in which each of the 4 measured parameters (size, protein, DNA, and RNA content) depend on growth rate. “At a given temperature, size and composition are found to depend in a simple manner on the growth rate afforded by the medium. This *implies* that media that give identical growth rates produce identical physiological states, *regardless of the actual constituents of the media*” [11] (emphasis added).

Based on analysis of cells cultured at different temperatures, Schaechter and colleagues further clarify that culture medium composition dictates growth rate, and it ultimately dictates the chemical composition of the cell. Despite this important caveat, growth rate—not medium composition—quickly became perceived as the primary driver of cell cycle progression [12] in part because of the simplicity with which it lends itself to mathematical modeling. This positive relationship between growth rate, cellular composition, and cell cycle progression is colloquially referred to as the “growth law” or “nutrient growth law” [11,13,14].

## The Cooper–Helmstetter model of cell cycle progression

Once SMK identified a positive connection between nutrient-imposed growth rate, cell size, and cell composition, the next challenge was to determine how this connection was achieved. Focusing on DNA replication and leveraging their ability to synchronize cells with their “baby machine,” Cooper and Helmstetter analyzed DNA synthesis in *E*. *coli* in real-time across 13 different media [15].

Incorporating data from Schaechter and colleagues [11] and work from Cairns [16] identifying the circular nature of the bacterial chromosome, Cooper and Helmstetter developed a quantitative phenomenological model of the *E*. *coli* cell cycle [1]. The Cooper–Helmstetter model posits 2 distinct replication regimes: single fork and multifork. Single fork (really single round) replication with up to 2 forks proceeding at a time prevails during slow growth, and the time required to complete a round of DNA replication (C-period) varies with nutrient-imposed population growth rate as does the period between the initiation of new rounds of DNA replication and the initiation of new rounds of cell division [1,15]. During the multifork regime, division and initiation continue to co-vary with population mass doubling time, however, elongation rate (and thus C period) plateaus [1] (Fig 1). Because of this imbalance, new rounds of replication are started prior to completion of the previous one providing an explanation for the multiple origins of replication observed by Cooper and Helmstetter and others, [1,15,17–22] including later studies that assess cell size [8,12,23–26].

## Population and single-cell data tell different stories

Technological limitations meant that Cooper–Helmstetter had to rely on population level data to develop their phenomenological model of the *E*. *coli* cell cycle. As they themselves note, their model applies specifically to idealized average cells. They intended to explain the phenomena of multifork replication as overlapping replication cycles, not to provide a mechanistic framework from which to understand relationships between cell cycle events [10].

**Fig 2:**
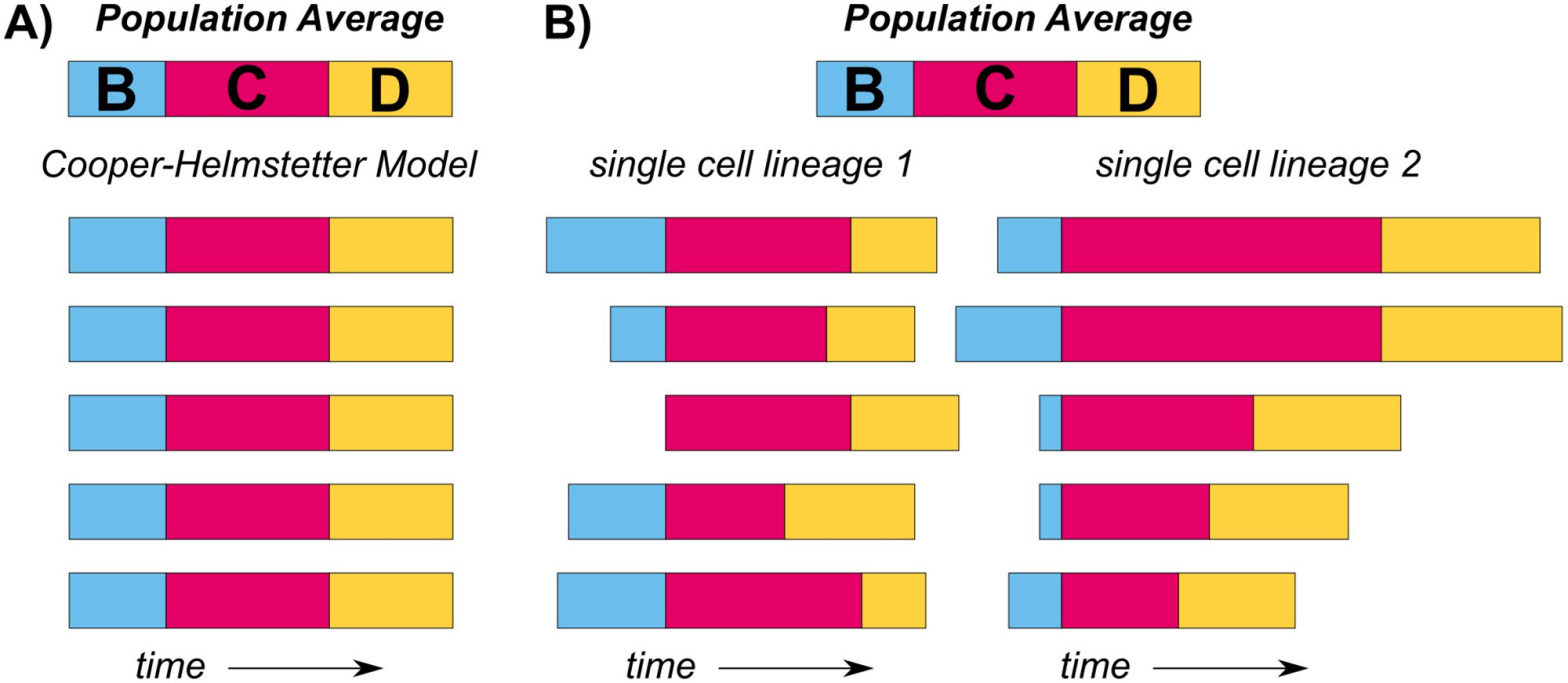
Deterministic paradigm vs. stochastic nature of cell cycle timescales. (A) The Cooper–Helmstetter model assumes that all cells within a given condition follow the population average B, C, and D periods. (B) Relative B, C, and D periods are shown over multiple consecutive replication cycles for 2 cell lineages grown on MOPS glucose (based on data from Si and colleagues). Significant differences between replication cycles necessitate a new theory accounting for stochasticity.

Individual cells do not behave like average idealized cells, however. In individual cells, stochasticity adds another layer of complexity to the already inherently complex process of overlapping cell cycles. Single-cell data reveal high levels of stochasticity regarding both growth rate and the temporal progression of cell cycle events (Fig 2). Even when the mass doubling time of a population is held constant, the instantaneous growth rate of single cells within that population varies as much as 3-fold [8,27,28] and elongation rates vary as much as 4-fold (250 to 1,000 nucleotides/second) [29–31].

Additionally, the “nutrient growth law” proposed by Schaechter and colleagues is inextricably linked to the Cooper–Helmstetter model, despite accumulating evidence that cell size and cell cycle progression can vary independently of nutrient-imposed growth rate [11,32–34]. DNA replication is inherently sensitive to medium composition as it directly impacts the availability of nucleotide precursors, and a coterie of mutations are known to impact size and/or DNA replication-independent of nutrient-imposed growth rate [32,34–37].

## An agnostic framework leveraging stochastic timers illuminates the relationship between nutrient composition and cell cycle progression in individual cells

The disconnects outlined above—the inability to account for stochasticity and the misassumption that growth rate is a primary driver of cellular physiology rather than medium composition—highlight the deficiencies of the Cooper–Helmstetter model as a universal tool for understanding the mechanisms underlying bacterial cell cycle control. To address this gap, we leveraged published single-cell datasets for slow and intermediate growth regimes [8] to develop an agnostic framework for medium-dependent stochastic bacterial cell cycle progression.

**Fig 3:**
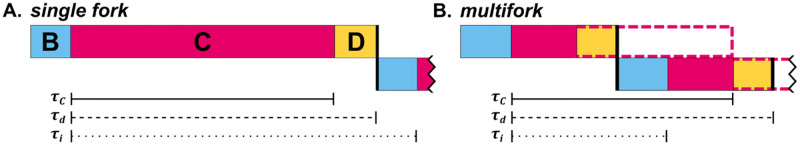
Stochastic timers. Visual representations of the timers *τ*_*i*_, ***τ***_***C***_, and *τ*_*d*_ in a cell undergoing single fork (A) and multifork (B) replication. These stochastic timers represent single-cell parameters. During multifork replication, C periods extend beyond a single division cycle. This overlap is indicated by extended dotted lines. Color schemes match Fig 2.

Tackling all problems listed above at once, our framework centers on the idea that at each initiation event, 3 new stochastic “timers”—corresponding respectively to single-cell inter-initiation time (the time between successive rounds of replication), ***τ***_***i***_; fork completion time (C period duration of individual cells), ***τ***_***C***_; and the time between initiation and the corresponding division event (C+D period for individual cells), ***τ***_***d***_—all begin to tick (Fig 3). Thus, the relative order of completion of these 3 timers determines the fork number at the start of the next replication cycle. We elected to begin with initiation rather than other cell cycle events as this step is traditionally viewed as the beginning of the bacterial cell cycle. In *E*. *coli*, replication initiation is tied to cell growth via accumulation of the initiator protein DnaA to threshold levels [8,38–43].

We observed that the distributions of the 3 single-cell variables (*τ*_*i*_, *τ*_*C*_, and *τ*_*d*_) generally vary with the stochastic single-cell exponential growth rate, ***k***, consistent with previous single-cell data [8,27,28]. Thus, we extract the stochastic distributions of these timers along with the subsequent replication cycle’s growth rate as experimentally measured functions of *k* (see S1 File for details), different for each independent growth condition.

To account for media-dependent variations in timer distributions, we generated “calibration curves” for each media condition and used these curved as input in our framework to simulate the next *τ*_*i*_, *τ*_*C*_, *τ*_*d*_, and *k* for each consecutive replication cycle (Fig A in S1 File). This framework is agnostic to the specific mechanisms governing the dependence on *k*, and thus robust to nutrient-dependent or strain-dependent differences in cell growth and cell cycle progression.

**Fig 4:**
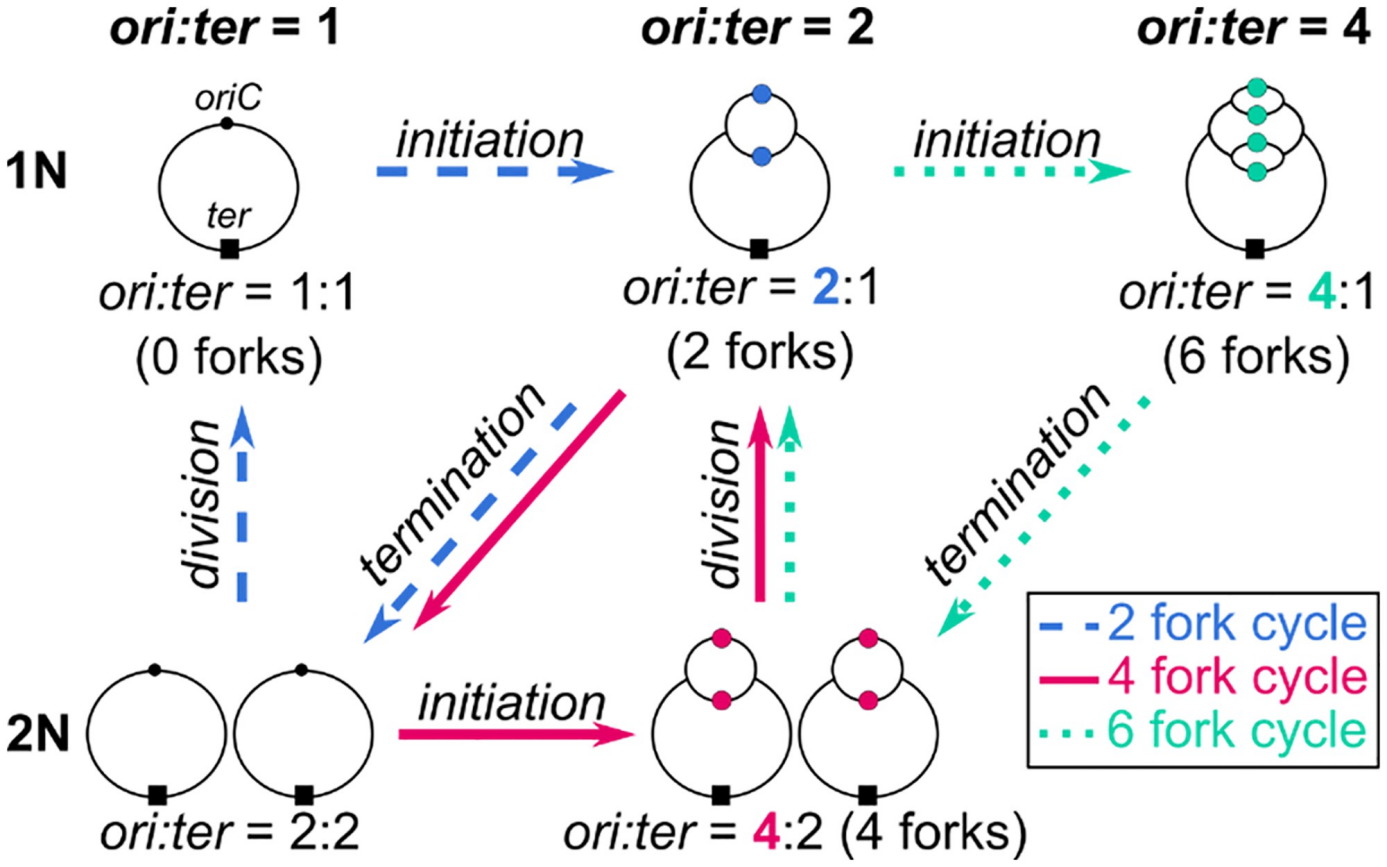
Flow chart depicting the possible replication cycles depending on the order of initiation, termination, and division events. *N* represents the number of chromosome copies present in the cell and is equal to the number of termini (*ter*, ■). The 2-fork cycle (blue) forms 2 forks upon initiation at *oriC*, ●, then replication is completed leaving 0 forks. The 4-fork cycle (magenta) progresses from 4 forks at initiation to 2 forks after division to termination. The 6-fork cycle (teal) progresses from 6 forks at initiation to 4 forks after termination of previous replication to 2 forks after division to 6 forks after new round of initiation. Further configurations with 8 forks or more are relevant only at growth rates faster than available in this dataset.

In slow growth conditions, M9 acetate (population mass doubling time 195 min), cells typically initiated and completed a single round of replication (single fork) per cell cycle (2-fork cycle in Fig 4, Cooper–Helmstetter model slow growth regime) [1,15,44]. Mathematically, the hierarchy between the 3 timers can be described as *τ*_*i*_ > *τ*_*d*_ > *τ*_*C*_. However, in some cases, after termination, we observed a new round of replication initiation prior to division (*τ*_*d*_ > *τ*_*i*_ > *τ*_*C*_), leading to a state with a total of 4 active replication forks divided across 2 chromosomes (*ori*:*ter* = 4:2, Fig 4). Distinguishing these cases does not depend on *τ*_*C*_, thus, under these conditions, our framework can be simplified to require input of only 2 timers as functions of *k*: (i) **inter-initiation time**, *τ*_*i*_; and (ii) **time to division**, *τ*_*d*_.

In intermediate conditions, MOPS glucose and MOPS glycerol 11aa (mass doubling times 52 min and 63 min, respectively), multifork replication occurred when a new round of replication was initiated prior to completion of the ongoing round of replication, resulting in 6 forks (*τ*_*d*_ > *τ*_*C*_ > *τ*_*i*_). In these media, single fork replication consisted solely of the 4-fork replication pattern described above in which *τ*_*d*_ > *τ*_*i*_ > *τ*_*C*_, corresponding to a situation in which a single round of replication terminated and reinitiated prior to division. These cases can be distinguished irrespective of *τ*_*d*_, simplifying our framework to only require *τ*_*i*_ and *τ*_*C*_ as inputs.

**Fig 5:**
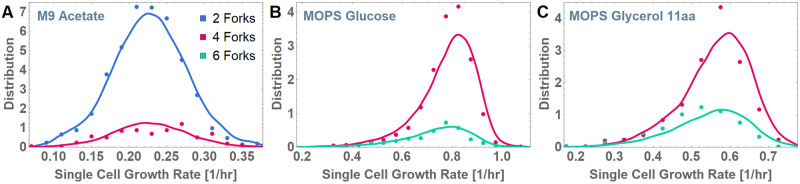
Comparing model predictions to experimental data. Model simulations (dashed lines) are compared with the experimentally obtained distributions (solid lines) of number of forks after initiation at different single-cell growth rates for the following growth conditions: (A) M9 acetate, (B) MOPS glucose, and (C) MOPS glycerol 11aa. Colors represent different number of forks after initiation: 2 (blue), 4 (magenta), and 6 (teal). There is a close match between model predictions and experimental data, indicating that the presence of different numbers of forks and both single and multifork replication within the same growth condition is purely a consequence of the inherent stochasticity in the 3 time periods governing the replication cycle (the C period, the inter-initiation period, and the time to division), each of which depends solely on single-cell growth rate for a given growth condition.

Using this streamlined version and the “calibration curves” obtained from the same single-cell dataset [8] (Fig A in S1 File), we simulated the population distribution of the number of replication forks per cell. The results of our fitting parameter-free simulations match experimental data extremely well, thus validating the soundness of the conceptual framework (Fig 5A).

Altogether, for these datasets our framework requires only *τ*_*i*_ and *τ*_*C*_ to differentiate between single and multifork replication independent of media composition. Notably, although the average population mass doubling time in MOPS glucose and MOPS glycerol 11aa are on either side of the Cooper–Helmstetter 60-min mass doubling time, we did not observe an abrupt plateau in single-cell C period (*τ*_*C*_) under either condition (Fig A in S1 File). It remains possible, however, that $\bar{\tau_{C}}$ (the population averaged value) may eventually plateau under more nutrient-rich conditions. From an unconstrained mathematical perspective of the population level, $\bar{\tau_{d}}$ is also expected to plateau as population mass doubling time is reduced, since *τ*_*d*_ is always greater than *τ*_*C*_, by definition (Fig 3). In contrast, due to the constraint that every division must be preceded by a corresponding initiation, $\bar{\tau_{i}}$ follows the same trend as mass doubling time when the nutrient-imposed growth rate is varied.

## Relationship between stochastic timers dictates replication fork number

Taken together, the measured and predicted timer behaviors suggest that multifork replication is a consequence of changes in the relationship between individual timers at fast single-cell growth rates. The timers differentially impact the relationships among cell cycle events depending on growth regime (i.e., slow, intermediate, or fast growth). During slow growth fork numbers are solely determined by the relative order of *τ*_*i*_ and *τ*_*d*_, while during intermediate growth they are determined by *τ*_*i*_ and *τ*_*C*_. During fast growth, we predict that all 3 timers play a role in determining fork numbers, especially during growth conditions that promote a mixture of allowable chromosome configurations and fork numbers.

**Fig 6:**
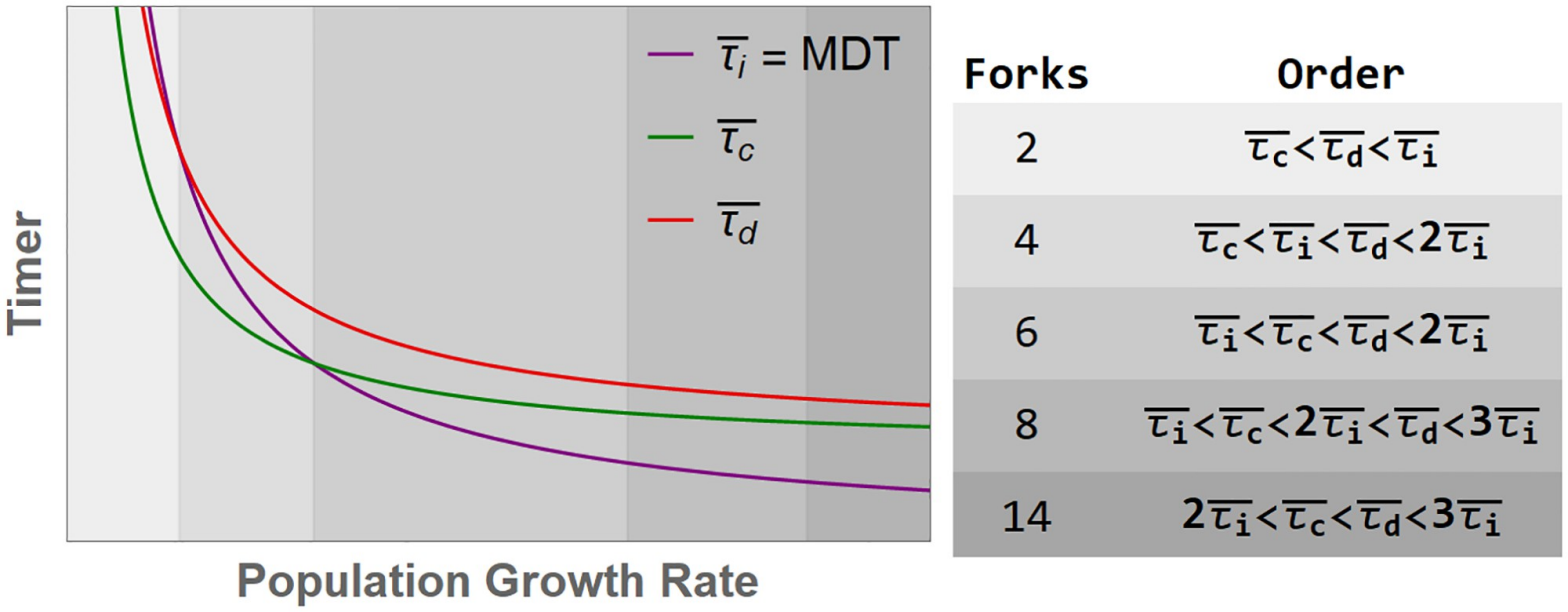
Determining dominant fork numbers from nutrient-imposed population growth rate. Our expectation for the trends in $\bar{\tau_{i}}$, $\bar{\tau_{C}}$, and $\bar{\tau_{d}}$ (representing the population mean values related to the stochastic timers *τ*_*i*_, *τ*_*C*_, and *τ*_*d*_) as a function of nutrient-imposed population growth rate. We expect mean *τ*_*C*_ and *τ*_*d*_ to flatten as growth rate goes to infinity, while mean *τ*_*i*_ (equal to mass doubling time) approaches zero. Fork numbers at different population growth rates are determined by the relative order of these timers, as shown.

At the population level, our framework predicts the emergence of 8-fork (or more) cycles (*τ*_*i*_<*τ*_*C*_<2*τ*_*i*_<*τ*_*d*_<3*τ*_*i*_) during fast growth, a mixture of 4-fork (*τ*_*C*_<*τ*_*i*_< *τ*_*d*_<2*τ*_*i*_) and 6-fork cycles (*τ*_*i*_<*τ*_*C*_< *τ*_*d*_<2*τ*_*i*_) during intermediate growth (near 60 min mass doubling time), and a combination of 2-fork and 4-fork cycles during slow growth. Supporting the validity of our framework, we obtained a close match with data from all 3 conditions without any fitting parameters (Fig 5B and 5C) and an updated and more detailed rendering of the Cooper–Helmstetter model (Fig 6 versus Fig 1).

## A roadmap for the application of this model to open biological questions

To test the ability of our agnostic framwork to address mechanism, we assessed the fork number-dependence of the divergent relationship between *τ*_*C*_ and single-cell growth rate. The clear divergence between single-cell growth rate and *τ*_*C*_ in fast-growing subpopulations suggests that replication elongation is negatively impacted in these cells. But why?

There are 3 major (but not only) explanations for the negative relationship between *τ*_*C*_, elongation rate, and mass doubling time in cells cultured at fast growth rates in nutrient-rich medium. In the first, the enzymatic activity of the replisome reaches maximum velocity in fast-growing cells. In the second, essential parts of the replication machinery or its substrate (e.g., dNTPs) become limiting at rapid growth rates (titration model). And in the third, replisomes begin to interfere with one another during higher order multifork replication, reducing average elongation rate through some form of steric interference (fork spacing model).

Although the first model is appealing, the idea that replisome efficiency reaches maximum velocity at population mass doubling times of 60 min or less is not well supported. Delaying the initiation of replication via mutations that reduce *E*. *coli* cell size [36] or by altering the accumulation or activity of the initiator protein DnaA, leads to significant (as much as 30%) reductions in C-period without a corresponding change in growth rate [45,46].

**Fig 7:**
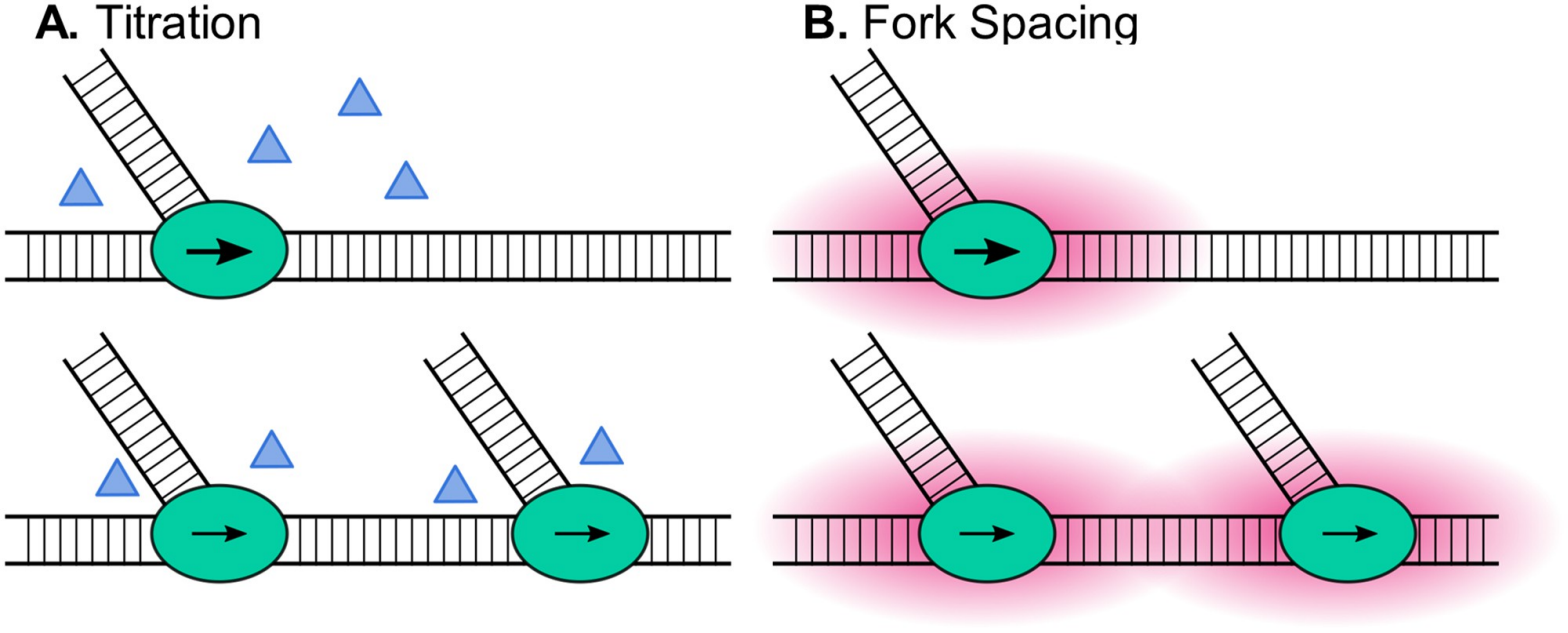
Two hypothesized models for C period decrease as growth rate increases. (A) Titration. (B) Fork spacing. Ovals represent active replisomes, triangles represent accessory replisome components and dNTP substrates, arrows represent relative replisome speed, and red clouds around the replisome represent steric repulsion and topological changes that alter replisome kinetics.

To distinguish between the remaining fork titration and fork spacing models, we plotted *τ*_*i*_ and *τ*_*C*_ relative to *k* in the 2 intermediate growth conditions and separated the population based on cycles containing either 4- or 6-fork cycles (Fig 4). A reduction in *τ*_*i*_ and *τ*_*C*_ (and consequently *τ*_*d*_) relative to *k* in a fast-growing population that is independent of total fork number would be consistent with titration of limiting replication substrates or enzymes (“titration model,” Fig 7A). Conversely, reductions in *τ*_*i*_ and *τ*_*C*_ that are correlated with fork number would support a model in which physical constraints decrease the maximum replication rate due to the increased number of replication forks progressing on a single strand (“fork spacing model,” Fig 7B).

**Fig 8:**
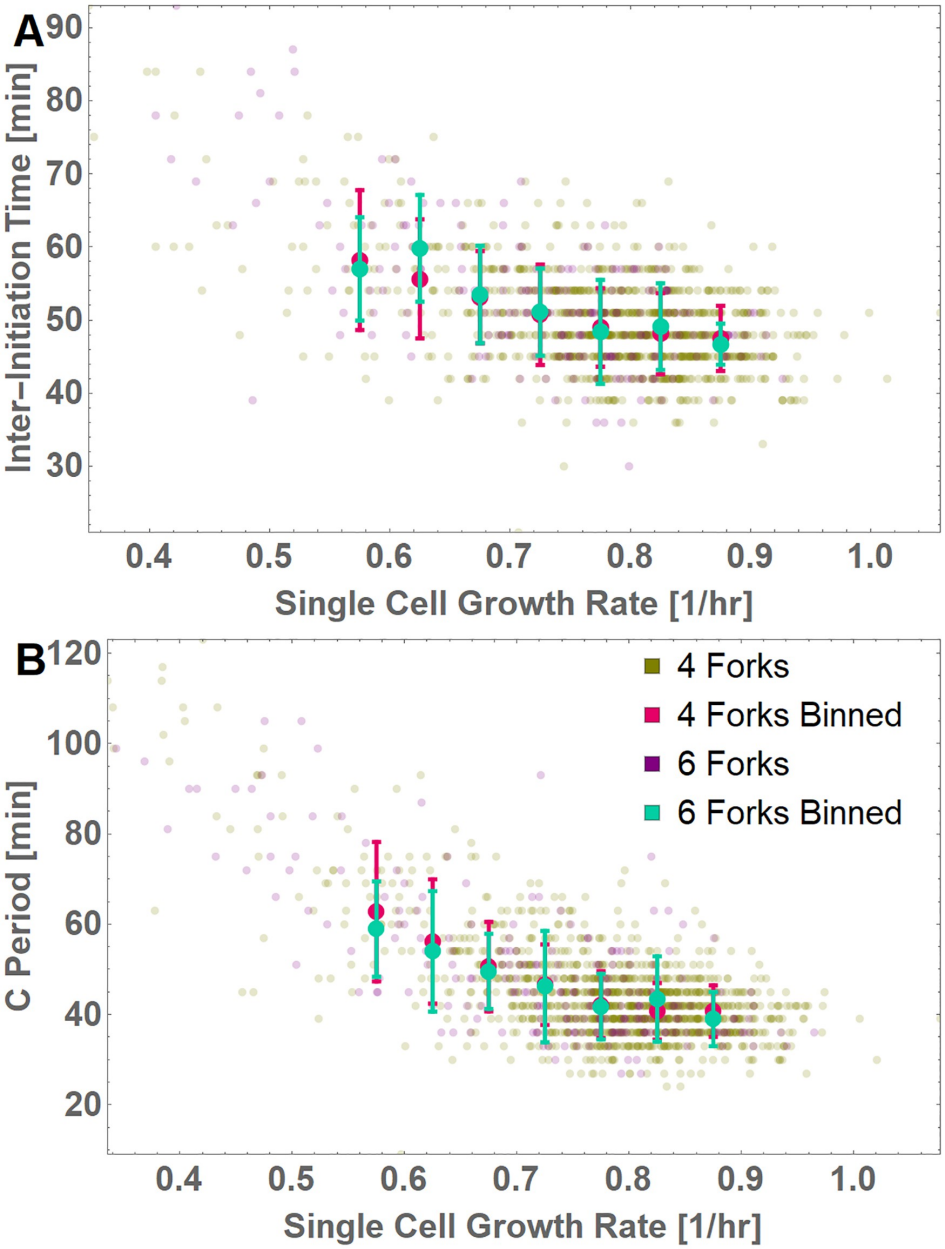
Both inter-initiation time and C period are independent of fork number. (A) Inter-initiation time (*τ*_*i*_) and (B) C period (*τ*_*C*_) is plotted against single-cell growth rate (*k*) for an intermediate growth condition (medium 4). Mean and SD of binned C periods and the spread of data points are plotted separately for ● 4-fork (single fork) and ● 6-fork (multifork) data, based on the fork number observed just after initiation.

Applying this “test,” we observed diminishing returns to *τ*_*C*_ as *k* increases, independent of the number of replication forks present in individual cells (Fig 8). Given the limited number of 4- and 6-fork cycles in the current dataset, our future work entails the full evaluation of this question with sufficiently large datasets representing a wider range of growth conditions. Then, we can fully dissect the mechanistic and molecular actors underlying this apparent fork independence.

## Discussion

Scientific progress depends on our ability to incorporate new information and is often driven by technological advancement. While the Cooper–Helmstetter model has served a valuable function—contextualizing and inspiring work on the bacterial cell cycle for over 50 years—rapid advances in single-cell analysis reveal its limitations.

To fill this gap, we developed a new framework with which to understand cell cycle coordination. This framework offers numerous advantages for the evaluation of single-cell data. Importantly, it may be applied to any strain in any growth medium in any growth regime. Our framework is agnostic to the mechanism underlying the stochastic dynamics of initiation, replication and division, and simply captures these dynamics through the experimentally measured “calibration curves” for each of these timers as functions of single-cell growth rates.

In sum, recognizing the value of single-cell data as a framework from which to understand the molecular mechanisms underlying cell cycle progression in bacterial cells is just the first step. Larger, more comprehensive single-cell datasets spanning a wide range of MDTs and media compositions is essential to determine the relationship between nutrient availability and cell cycle progression at high resolution not only in *E*. *coli* but also in other bacteria, model, and non-model alike. We look forward to the next chapter!

## Supporting information

S1 File The supporting information file details the protocol for generating calibration curves from data and includes a figure in which the calibration curves used as inputs in the model for different growth conditions are plotted. (PDF)
