# Supplementary material for: Beyond the average: An updated framework for understanding the relationship between cell growth, DNA replication, and division in a bacterial system
Source: PLoS Genet. 2023 Jan 5;19(1):e1010505. doi: 10.1371/journal.pgen.1010505 (PMC9815640; doi:10.1371/journal.pgen.1010505)
Supplement: S1 File — (PDF) [file pgen.1010505.s001.pdf]

Supporting Information for
Beyond the average: An updated framework for understanding the relationship between cell growth, DNA replication, and division in a bacterial system

Sara Sanders[1 †], Kunaal Joshi[2 †], Petra Anne Levin[1 *], and Srividya Iyer-Biswas[2,3 *]

[1]Department of Biology, Washington University in St. Louis, St. Louis, Missouri, USA
[2]Department of Physics and Astronomy, Purdue University, West Lafayette, Indiana, USA
[3]Santa Fe Institute, Santa Fe, New Mexico, USA

† Equal contributions
* plevin@wustl.edu (PAL) and iyerbiswas@purdue.edu (SIB)

Protocol for generating "calibration curves" from data

We tested our model on raw data published by the Jun lab (originally generated to study the validity of the independent double adder model of size control) [8] spanning three growth conditions: M9 acetate (mass doubling time 195 min, equivalent to Cooper-Helmstetter's slow, single fork regime), and MOPS glucose and MOPS glycerol 11aa (MDTs 52 min and 63 min, respectively, both near the transition between Cooper-Helmstetter's single fork and multifork regimes).

To express the functional dependencies of the distributions of the three timers τ_i, τ_C, and τ_d on single cell growth rate k in a form usable by our model, we identified emergent simplicities, or scaling collapses, and generated calibration curves for each media condition. Briefly, for each of the three growth conditions, we plotted all data points for the given condition relative to the single-cell exponential growth rate. We then subtracted the best-fit mean at the corresponding growth rate from each data point and divided the resulting value by the best-fit standard deviation (S.D.) at that growth rate. Finally, we measured the probability distribution of these scaled values, ***F***. Once again, the use of distinct calibration curves generated for each condition highlights the important relationship between medium composition and cell cycle dynamics, and reinforces previous work identifying the limitations of population "growth rate" as an independent variable with direct impacts on cell size and other phenotypes.[32–34]

$$P(x|k) = \frac{1}{\sigma_{x|k}} F\left(\frac{x-\langle x|k\rangle}{\sigma_{x|k}}\right) \qquad \textbf{(Eq. 1)}$$

Within experimental error, the growth rate-independent distribution *F* can be appropriately scaled as shown in **Eq. 1** to give the conditional distribution of the stochastic timescale in question at any given growth rate, with the appropriate mean and variance, and approximate correct shape. Here, x is the stochastic timescale in question; $\sigma_{x|k}$ and $\langle x|k\rangle$ are the fitted S.D. and mean, respectively, as functions of k. (See **Fig AA, AB, AD, AE, AG, AH in S1 file**) We performed a similar analysis to characterize the dependence of k during a given inter-initiation cycle on its value during the previous cycle (**Fig AC, AF, AI in S1 file**). Together, these experiment-derived calibration curves serve as inputs in our model and are used in our simulations to generate the next τ_i, τ_C, τ_d, and k for each consecutive replication cycle.

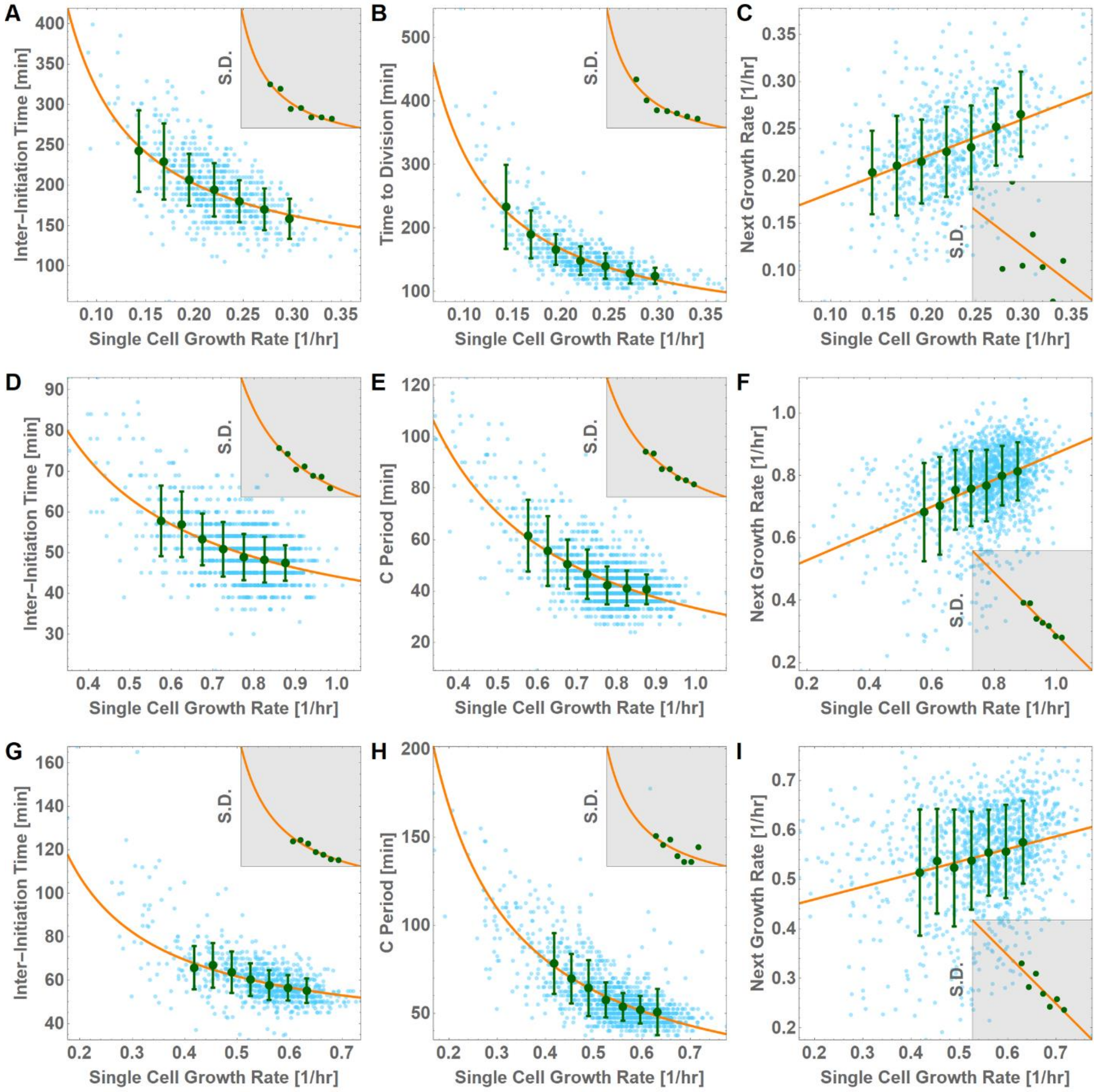

Fig. A. Calibration curves used as inputs in the model. For cells growing in (A-C) M9 Acetate, (D-F) MOPS Glucose, and (G-I) MOPS Glycerol 11aa, data points with binned mean and S.D. of (A, D, G) inter-initiation time, (B) Time to Division in slow growth condition and (E, H) C Period in intermediate growth conditions (as explained in the main text, intermediate growth conditions require C Period instead), and (C, F, I) single-cell growth rate of subsequent generation are plotted as a function of single-cell growth rate of current generation (inset: S.D. as a function of growth rate for the corresponding binned points). The orange line is the best-fit line (A and B: y=a+b/x and C: y=a+bx) fit using the binned mean. The observation that each binned mean and S.D. are quantitatively related enables us to use Eq. 1 to represent the different distributions at different single-cell growth rates within a growth condition by the same invariant rescaled distribution. This results in a significant simplification of model complexity. Orange best fit curves correspond to the mean and S.D. of these quantities, which we take as inputs to our model when using the rescaled distribution formulation; thus, their match with the green binned experimental data points is essential for accuracy of our results. Note that the S.D. in (C) inset for M9 Acetate is almost constant (with a variation of only 8%).
